# Multi-Omics Characterization of the Spontaneous Mesenchymal–Epithelial Transition in the PMC42 Breast Cancer Cell Lines

**DOI:** 10.3390/jcm8081253

**Published:** 2019-08-19

**Authors:** Sugandha Bhatia, James Monkman, Tony Blick, Pascal HG Duijf, Shivashankar H. Nagaraj, Erik W. Thompson

**Affiliations:** 1 Institute of Health and Biomedical Innovation, Queensland University of Technology, Brisbane, QLD 4059, Australia; 2 School of Biomedical Sciences, Faculty of Health, Queensland University of Technology, Brisbane, QLD 4000, Australia; 3 Translational Research Institute, Brisbane, QLD 4102, Australia; 4 University of Queensland Diamantina Institute, The University of Queensland, Woolloongabba, QLD 4102, Australia

**Keywords:** copy number variations (CNV), epithelial–mesenchymal transition (EMT), karyotyping, mesenchymal–epithelial transition (MET), metabolism, proteomics, RNA-sequencing, seahorse extracellular flux analyser, whole exome sequencing

## Abstract

Epithelial–mesenchymal plasticity (EMP), encompassing epithelial–mesenchymal transition (EMT) and mesenchymal–epithelial transition (MET), are considered critical events for cancer metastasis. We investigated chromosomal heterogeneity and chromosomal instability (CIN) profiles of two sister PMC42 breast cancer (BC) cell lines to assess the relationship between their karyotypes and EMP phenotypic plasticity. Karyotyping by GTG banding and exome sequencing were aligned with SWATH quantitative proteomics and existing RNA-sequencing data from the two PMC42 cell lines; the mesenchymal, parental PMC42-ET cell line and the spontaneously epithelially shifted PMC42-LA daughter cell line. These morphologically distinct PMC42 cell lines were also compared with five other BC cell lines (MDA-MB-231, SUM-159, T47D, MCF-7 and MDA-MB-468) for their expression of EMP and cell surface markers, and stemness and metabolic profiles. The findings suggest that the epithelially shifted cell line has a significantly altered ploidy of chromosomes 3 and 13, which is reflected in their transcriptomic and proteomic expression profiles. Loss of the TGFβR2 gene from chromosome 3 in the epithelial daughter cell line inhibits its EMT induction by TGF-β stimulus. Thus, integrative ‘omics’ characterization established that the PMC42 system is a relevant MET model and provides insights into the regulation of phenotypic plasticity in breast cancer.

## 1. Introduction

Breast cancer, a leading cause of cancer death in women, is recognized as a molecularly heterogeneous disease [1,2]. Breast cancer cell culture systems and patient-derived xenografts (PDX) recapitulate many of the intrinsic molecular subtypes of breast cancer [3]. Various experimental techniques have been employed in these models, such as exome sequencing, copy-number analysis, whole-transcriptome, epigenome and methylome analyses, and identification of biomarkers that were mapped simultaneously to the genotypic and/or phenotypic behaviour in relevance to breast cancer subtypes or mammary gland development [4,5,6,7,8,9,10,11,12,13,14,15]. The compendium of molecular profiles defining up to 90 different breast cancer cell lines provides a valuable resource and has been studied extensively [10,11,12,13,15], not only for the discovery of new breast cancer genes [10], but also for investigations of their subtype-specific pathobiology, cancer stem cell biology, biomarkers, and response to different drug therapies [16]. Thus, cell culture systems provide important models for studying the molecular mechanisms of neoplastic transformation and are extremely useful in translational research.

Epithelial–mesenchymal plasticity (EMP), which encompasses epithelial–mesenchymal transition (EMT) and its reversal (mesenchymal–epithelial transition; MET), including hybrid states within this spectrum, is considered an important hallmark of cancer that drives metastasis [17,18,19]. EMT provides carcinoma cells with the ability to undergo cellular morphogenesis, allowing clusters of epithelial cells to form independent motile mesenchymal-like cells that can disseminate. MET is thought to subsequently reinstate proliferation and allow collective outgrowth at the metastatic site. The genetic and phenotypic heterogeneity of carcinoma cells across the EMP axis also has importance in therapy-resistance seen after EMT [20,21,22,23]. The molecular and cellular analyses of EMT features in breast cancer cell lines have also been analysed thoroughly and scoring tools have been developed to quantify the extent of EMT [24,25,26,27,28,29].

EMP studies in cancer cell lines have been widely reported. Therein, EMP is either induced by external stimuli, such as hypoxia [30], TGF-β [31], EGF [32], FGF [33], or by inducible expression or repression of EMT-inducing transcription factors [34,35]. Transformed breast cancer cells obtained by introducing oncogenes or cancer-associated genes into normal primary human mammary epithelial cells, such as HMLE and D492 cells, have also been remodelled as HMLER and D492M, respectively, to study cancer development including EMT [36,37,38,39]. To our knowledge, the PMC42 system is the only breast cancer cell line model where a spontaneous MET event has led to a new stable variant [40]. In this regard, the PMC42 system provides a unique case study to investigate the molecular and phenotypic characteristics of a stable MET in carcinoma without the use of external stimuli or genetic manipulation.

The relationships between different levels of regulation (genomic, transcriptomic, metabolomic, etc.) in the intrinsic endogenous plasticity (i.e., not exogenously induced) seen in several EMP systems has not been systematically studied, although each has separately been implicated in EMP regulation. Here, we interrogate and integrate the molecular portrait of PMC42, a unique human breast carcinoma cell line originally established from the pleural effusion of a breast cancer patient in 1983 [41]. Original cultures of PMC42 cells were reported to be heterogeneous, with at least eight different morphological types identified by phase contrast and electron microscopy [41,42]. In earlier studies, PMC42 cells were shown to grow both as a monolayer and as cords in suspension, and have shown features of myoepithelial cells [41,43,44]. PMC42 cells obtained from Dr. Robert Whitehead [41,42] were subsequently annotated as PMC42-ET. PMC42-ET exhibits a ‘Basal B’ (mesenchymal) transcriptome (E Tomaskovic-Crook and T Blick, unpublished observation) [30] and PMC42-LA cells were a spontaneous derivative cell line from PMC42-ET developed by Dr. Leigh Ackland [40,45]. Although epithelially shifted to the extent that they can produce a functional epithelium in 3-dimensional (3D) cultures [45], they still cluster transcriptomically with PMC42-ET cells (E Tomaskovic-Crook and T Blick, unpublished observation) [30]. This makes these cell lines an ideal system to study MET. Comparison of the karyotype, exome, transcriptome, proteome and metabolic phenotype of PMC42-ET and its epithelial “daughter” cell line PMC42-LA were performed to gain insights into the dynamic events that contributed to this spontaneous MET, in reference to EMP studies.

## 2. Materials and Methods

### 2.1. Cell Lines and Cell Culture

Cells (BT-549, Hs578T, MCF-7, T47D, MDA-MB-157, MDA-MB-231, MDA-MB-468, SUM-159) were obtained from the American Type Culture Collection (Manassas, VA, USA). PMC42-ET and the derivative PMC42-LA cell line were derived from a breast cancer pleural effusion with appropriate institutional ethics clearance (Institutional Review Board of the Peter MacCallum Hospital, Melbourne) [41,42,43,45]. 

Cell lines were cultured in Dulbecco’s modified Eagle’s medium (DMEM) containing glucose (4.5 g/L), L-Glutamine (0.5 g/L) and sodium pyruvate (0.1 g/L) (Corning, Catalog number 10-013-CVR), and supplemented with 10% foetal bovine serum (FBS; Gibco^TM^, Thermo Fischer Scientific, Waltham, MA, USA) and antibiotics penicillin and streptomycin (Gibco^TM^, Life Technologies, NY, USA; Catalog number 15140122), and maintained at 37 °C, 5% CO_2_, and 95% humidity. Cells were routinely tested and were negative for *Mycoplasma*.

### 2.2. Preparation of Metaphase Spread and Karyotyping

After 60%–70% of confluency was achieved in 60 mm dishes, cells were treated with 10 μL of demecolcine (10 μg/mL) for 3–4 h. Cells were harvested using trypsin (Corning™ 25053CI) and the pellet was gently treated with hypotonic solution (75 mM KCl) for 40–60 min at 37 °C and fixed in cold methanol/acetic acid (3:1). Two or three drops of suspended cells were applied to glass slides and chromosomes were stained with DAPI and imaged by confocal microscopy (Olympus Fluoview FV1200 Confocal Laser Scanning Microscope, Olympus Australia Pty. Ltd., Melbourne, Victoria, Australia). Counting was performed manually in ImageJ. Karyotype assessment via G-band analysis was performed by a commercial genotyping service (StemCore Facility, Brisbane, QLD, Australia) and 50 metaphases were analysed per cell line.

### 2.3. DNA Extraction, Whole Exome Sequencing and Processing of Sequencing Data

Genomic DNA was extracted from cells using Bioline Isolate II Genomic DNA kit (Cat: BIO-52067) as per the manufacturer’s instructions. After quantifying the DNA and checking the purity, DNA samples were shipped to GENEWIZ (Suzhou, China) for whole exome sequencing (WES) and subsequent analysis. 

Genewiz, Inc. (Suzhou, China) performed initial quality control assessments and subsequent exome capture using the SureSelectXT HS Target enrichment kit (Agilent). All samples were paired-end multiplex sequenced (2 × 150 base pairs) on the Illumina Hiseq 2500 platform to a median target depth of over 50× WES data have been deposited at NCBI under BioProject ID PRJNA557326.

Paired-end reads underwent quality control before alignment to the reference human genome (hg19) using Burrows-Wheeler alignment (BWA, version 0.7.12-r1039) [46] and SAMtools (version 1.6) [47]. Realignment and recalibration were performed using the Genome Analysis Toolkit (GATK, version 3.5) [48]. Single nucleotide variants (SNVs) and insertions and deletions (indels) were called using GATK with default settings. Annotation of variants (SNP and indels) was performed using ANNOVAR [49,50]. Control-FREEC v 10.6 was used for detecting and filtering the CNV [51]. 

CRAVAT (Cancer-Related Analysis of Variants Toolkit), a tool specifically tailored to analyze cancer-specific variants [52], was used for identification and prioritization of genes with a possible role in cancer tumorigenesis in the PMC42 cell lines. Identification and annotation of cancer-specific driver missense mutations was performed using CHASM (Cancer-specific High-throughput Annotation of Somatic Mutations) [53,54]. To identify and prioritize pathogenic missense mutations, VEST (Variant Effect Scoring Tool), a supervised machine learning-based classifier [55], was also applied. Both CHASM and VEST computational scores are integrated in the CRAVAT suite. 

### 2.4. RNA Extraction, cDNA Synthesis and RT-qPCR 

Total RNA was extracted from cells using TRIzol (Life Technologies) and subsequent reactions were carried out as per the Bioline Isolate II RNA Micro kit manufacturer’s instructions, and cDNA was synthesized using SensiFAST^TM^ cDNA Synthesis kit from Bioline. Real time-quantitative PCR (RT-qPCR) was performed using the SYBR Green Master Mix in a ViiA7 Real-Time PCR system (Applied Biosystems) and analysis performed using the Quantstudio^TM^ Real-Time PCR software v1.1 (Applied Biosystems, Life Technologies). The primers sequences are listed in Supplementary Table S1. Hierarchical clustering of transcriptional profiles was performed using the Morpheus online tool [56]. Data were normalized against the overall mean expression of all measured genes [57]. 

### 2.5. Whole Transcriptome Sequencing and Analysis of PMC42 Cell Lines

mRNA transcript abundances for PMC42 cell lines were measured using RNA-seq as previously reported [30]; data have been deposited at NCBI under bioproject ID PRJNA322427. Sequence alignment to the hg19 reference genome was performed using TopHat [58] with default parameters. Differential expression analysis was performed using CuffDiff [58] with default parameters. RNA sequencing results were re-analysed for interrogation of fold changes across the two PMC42 cell lines with respect to the chromosome number. Gene Set Enrichment Analysis [59] was also applied to identify enrichment of gene signatures contained in the Molecular Signatures Database (MSigDB). The filtered gene lists with *p* < 0.01 were also examined by Ingenuity Pathway Analysis® (IPA) for functional annotation and gene network analysis. The GSVA method from the GSVA R/Bioconductor package was also applied on the gene expression data for the PMC42-ET and PMC42-LA cell lines to score samples against the TGFβ-EMT signature.

### 2.6. Data-Independent Acquisition (DIA) Mass Spectrometry of PMC42 Cell Lines

Cells were washed with ice-cold phosphate buffered saline (PBS), and lysed directly in cell lysis buffer containing 4% (w/v) SDS, 10 mM dithiothreitol (DTT), 10 mM Tris-HCl along with Roche compete protease and phosphatase inhibitors (Roche, Rotkreuz, Switzerland). Lysates were sonicated to shear DNA, and protein concentration was quantified using the Pierce™ BCA Protein Assay Kit (Thermo Scientific, Rockford, IL, USA). On the basis of protein quantifications, each experimental sample was aliquoted into 25 μg samples for processing using the FASP method [60]. Digestion was performed overnight using Trypsin/Lys-C (Promega) mix in 1:50 of protein. Fragmented peptides were then dissolved in 0.1% formic acid and processed for a final clean-up step using C18 Zip-Tips (Millipore; Billerica, MA, USA). 

Protein Pilot (V 4.1) software from SCIEX was used for peptide identification. The human protein library was built using the UniProt database (release 2018_05, [61]) with the following settings: Sample Type, identification; Cysteine alkylation, acrylamide; Instrument, TripleT of 5600; Species, human; ID focus, Biological modification; Enzyme, trypsin; Search effort, thorough ID. False discovery rate (FDR) was calculated within ProteinPilot software and peptides identified with greater than 99% and a local FDR of 1% was applied for the peptide identification. PeakView Software was employed to measure the peptide abundance with standard parameters [62] and manual inspection was carried out to confirm the accuracy of the spectra. Six peptides per protein were used to measure the protein abundance. The differences in protein abundance between PMC42-ET and -LA were calculated based on the significance and fold-changes. MSstats was used to calculate protein level significance by applying a linear mixed-effects model [63]. The model combines quantitative measures for a targeted protein across peptides, charge states, transitions, samples, and conditions; the system detects proteins that change in abundance among conditions more systematically than would be expected by random chance, while controlling the FDR. In house scripts in Python and R were developed for further analysis.

### 2.7. Fluorescence Activated Cell Sorting (FACS)

Cells were lifted with Accutase® (Corning, Catalog # 25-058-CI) and stained with anti-human CD44-FITC (BD Pharmingen) and anti-human CD24-PB (Exbio) antibodies at manufacturer’s recommended dilutions in 0.1% BSA (Bovine serum albumin, Sigma) diluted in DPBS for 1 h in a rotary shaker at room temperature. Cells were analysed in the presence of propidium iodide (1 µg/mL) using a BD LSRFortessa (BD Biosciences). After doublet discrimination and compensation for spectral overlap, data were analysed by using FlowJo Software (BD Biosciences). For TGFβR2 surface expression, cells were stained with primary antibody (RandD Systems, Cat# AF-241-NA) as per manufacture recommended dilutions for 1 h and then with secondary goat antibody for 1 h.

### 2.8. Immunocytochemistry

The cell lines were seeded at a density of 10,000 cells/well in 48-well plates (Thermo Scientific Nunclon^TM^ Delta Surface-150687). During immunocytochemistry, the growth medium was discarded, and cells were washed thrice gently with Dulbecco’s modified PBS (DPBS; pH 7.5). Briefly, cells were fixed in 4% paraformaldehyde ± 0.1% Triton X-100 (depending on the desired permeabilization conditions), rinsed with DPBS, and incubated with primary antibodies at 4 °C overnight. After rinsing again in DPBS, cells were incubated with an appropriate fluorescence-conjugated secondary antibody (Supplementary Table S2) and with diamidino phenyl indole (DAPI) as a nuclear stain (diluted to a final concentration of 1 µg/mL) for 2 h at room temperature in the dark with gentle rotary shaking. The plates were then washed thrice with DPBS and images were captured on a high-content imaging platform (Cytell Cell Imaging System (GE Healthcare) or IN Cell Analyser 6000 (GE Healthcare, Buckinghamshire, UK), as indicated), with approximately 6–9 fields of view taken per well. Images were further analysed and quantified using the IN Cell Investigator software v1.0 (GE Healthcare). 

### 2.9. Seahorse Metabolic Analyser

Collagen (Rat tail, type 1, Cat 354236, BD Biosciences)-coated Seahorse cell culture plates (Seahorse *Bioscience*, 102601-100) were seeded at a density of 20,000 cells per well (XFe96 cell culture microplate; Seahorse Biosciences, North Billerica, MA, USA). The cells were allowed to grow for 24–48 h at 37 °C in 5% CO_2_, after which the cells were washed and replaced with assay media (unbuffered DMEM or RPMI supplemented with 10 mM glucose, 1 mM sodium pyruvate, 2mM L-glutamine, no sodium bicarbonate at pH 7.4). The cells were incubated for 1 h at 37 °C in a non-CO_2_ incubator. Mitochondrial complex inhibitors (1.2 µM oligomycin, 1.2 µM carbonyl cyanide p-(trifluoromethoxyl)-phenyl-hydrazone (FCCP) and combined 1 µM rotenone with 1 µM antimycin A) were preloaded in the injection ports. For basal rate measurements, ECAR (Extra Cellular Acidification Rate) and OCR (Oxygen Consumption Rate) measurements were assessed. Experiments were performed in triplicates and the data were normalized by cell number. 

### 2.10. Statistical Analysis

All experiments were carried out at least three times unless otherwise indicated. Data were analysed using GraphPad Prism version 7 statistical software (GraphPad Software, La Jolla, CA, USA).

## 3. Results

### 3.1. Comparison of PMC42 Cell Lines with Other BC Cell Lines (Luminal, Basal A and Basal B)

**Figure 1:**
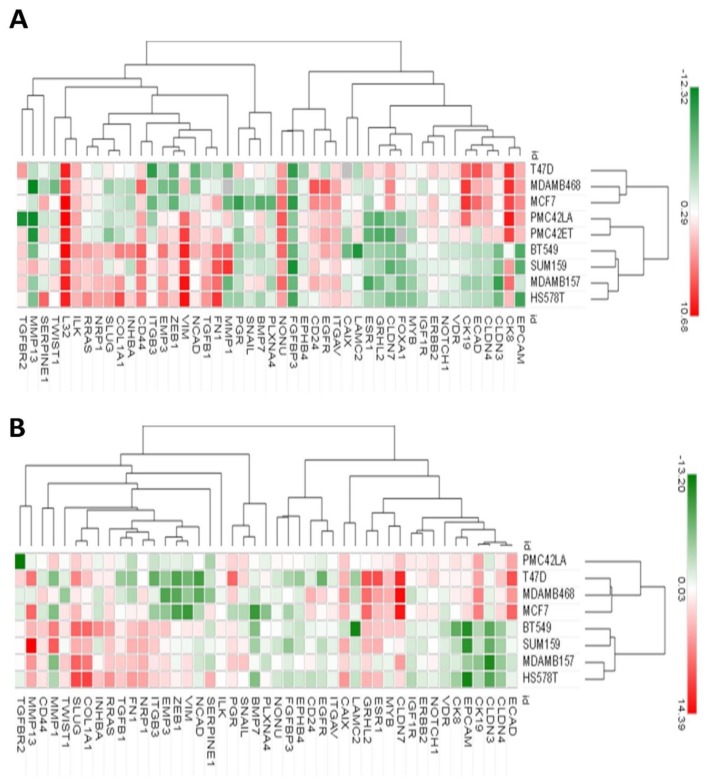
Gene expression heatmaps of selected breast cancer cell lines. (**A**) The normalized mRNA expression values of the breast cancer cell lines obtained against the overall mean expression of all measured genes were subjected to unsupervised hierarchical clustering using Morpheus (Gene-E tool). In the average-linkage cluster algorithm, Pearson correlation was used to measure dissimilarity. (**B**) Unsupervised cluster analysis of the relative expression values of breast cancer cell lines with respect to PMC42-ET.

Hierarchical clustering of RT-qPCR data of the 9 breast cell lines for EMP markers, inducers and regulators, along with the BC clinically relevant *ESR1*, *PGR* and *ERBB2* gene products, showed substantial variation across the cell lines and revealed two major branches (Figure 1A). Luminal cell lines MCF-7 and T47D, along with the Basal A MDA-MB-468 cell line formed one cluster, whereas the PMC42 cell lines were more closely associated with the other cluster of four Basal B cell lines (BT-549, SUM-159, MDA-MB-157, HS578T). Interestingly, when the heat map was computed on the basis of log_2_ fold difference in expression of each gene with respect to PMC42-ET cells, the PMC42-LA cell line clustered more closely with luminal MCF-7, T47D cells and Basal A MDA-MB-468 cells (Figure 1B). This further illustrates that PMC42-LA has more predominant epithelial markers as compared to its parental PMC42-ET cell line. Interestingly, we also observed the complete absence of expression of the FoxA1 gene in PMC42-ET cell line. 

### 3.2. CD44^+^CD24^−/low^ Phenotype Association with Breast Molecular Subtypes and Other EMT Markers

**Figure 2:**
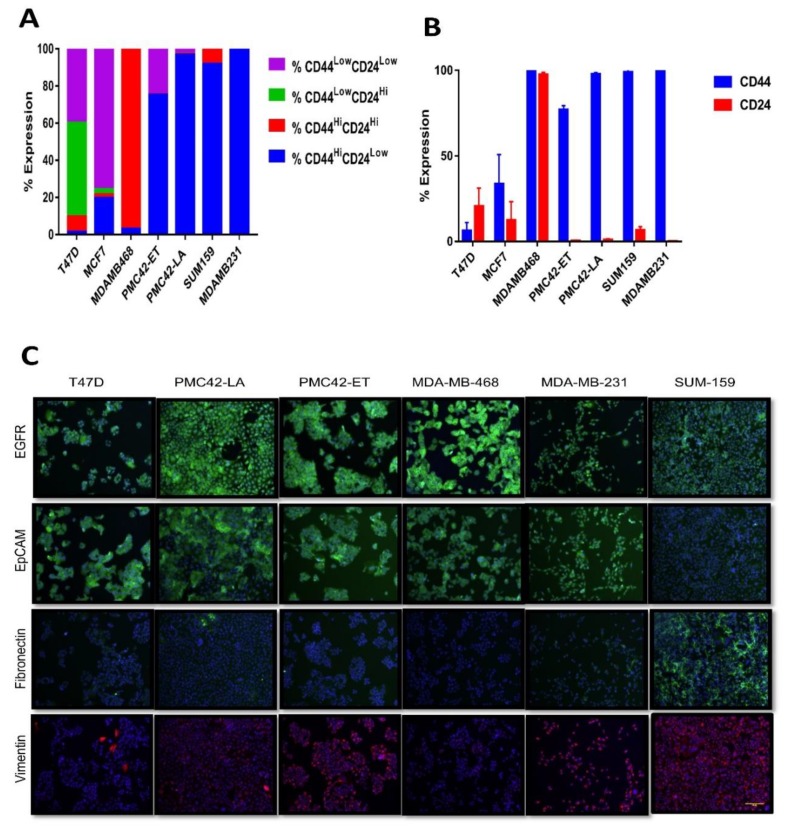
Assessment of stemness and other EMP markers. (**A**) Proportions of the subpopulations defined by the combination of the stem cell markers CD44 and CD24 in PMC42 cell lines and a panel of breast cancer cell lines. (**B**) The relative expression of the stem cell markers CD44 and CD24 in a panel of breast cancer cell lines representative of different molecular subtypes by flow cytometry. Mean ± SD of three independent experiments is shown. (**C**) Immunofluorescence microscopy analysis of EMT marker proteins. Cell lines were stained with antibodies against the epithelial marker EpCAM and against the mesenchymal markers EGFR, vimentin and fibronectin. Scale bar, 100 μm.

The CD44^high^/CD24^low^ profile is a putative marker of cancer stemness and is also associated with EMT phenotype [25]. The proportions of CD44^+/high^/CD24^−/low^ cell populations across the PMC42 cell lines were compared with five other breast cancer cell lines (MCF-7, T47D, MDA-MB-468, MDA-MB-231 and SUM-159) and the expression of these surface markers was simultaneously assessed by FACS analysis (Figure 2A,B). Surprisingly, PMC42-LA cells were remarkably stem-like in relation to these markers, as predominantly all cells gated within the CD44^high^/CD24^low^ subpopulation, similar to MDA-MB-231 and SUM-159 cells representing the most mesenchymal Basal-B subgroup [25], whereas 75.2% of the more mesenchymal PMC42-ET cells were in the CD44^high^/CD24^low^ state. In agreement with previous reports [64], MCF-7 cultures (Luminal subgroup; [25]), had a small population of CD24^−/low^/CD44^+^ cells (~20%) and the MDA-MB-468 cell line (Basal-A subgroup; [25]), had higher expression of both CD44 and CD24 markers (96.4% of cells gated in CD44^high^/CD24^high^ state). Compared to MCF-7 and T47D, all other cell lines were mainly constituted by cells with high levels of CD44, and except MDA-MB-468 also showed lower proportions of CD24 expression, consistent with their mesenchymal subgrouping (Figure 2A,B). 

The CD44^+/high^/CD24^−/low^ cell populations across PMC42-ET, PMC42-LA, MCF-7 and MDA-MB-468 were also assessed after stimulation of the cells with EGF or TGF-β for 72 h. Both MCF-7 and MDA-MB-468 cell lines exhibited a 10%–20% increase in their CD44^high^/CD24^low^ proportions after treatment with EGF, but not with TGF-β. PMC42-ET cells, however, could be made potentially stem-enriched with 93%–94% population of cells in CD44^high^/CD24^low^ state after stimulation with either EGF or TGF-β, which was due to increased CD44 expression (Supplementary Figure S1). 

EMT-associated markers EGFR, EpCAM, fibronectin and vimentin were also evaluated using immunofluorescence for the six breast cancer cell lines (Figure 2C). EGFR expression was highest for MDA-MB-468 cells, in which EGFR is known to be amplified [65]. EpCAM expression at cellular junctions was observed for T47D, PMC42-LA and MDA-MB-468 cell lines, and fibronectin expression was only observed for SUM-159 cells. Vimentin expression was universally positive in SUM-159, MDA-MB-231 and PMC42-ET cells, whereas on an average 10% and 20% of the cells were positive for vimentin expression in MDA-MB-468 and PMC42-LA cells, respectively, as quantified using IN Cell Investigator software, which is consistent with previous reports [39,40,66].

### 3.3. Comparative RNA-seq Analysis of PMC42 Cell Lines

**Figure 3:**
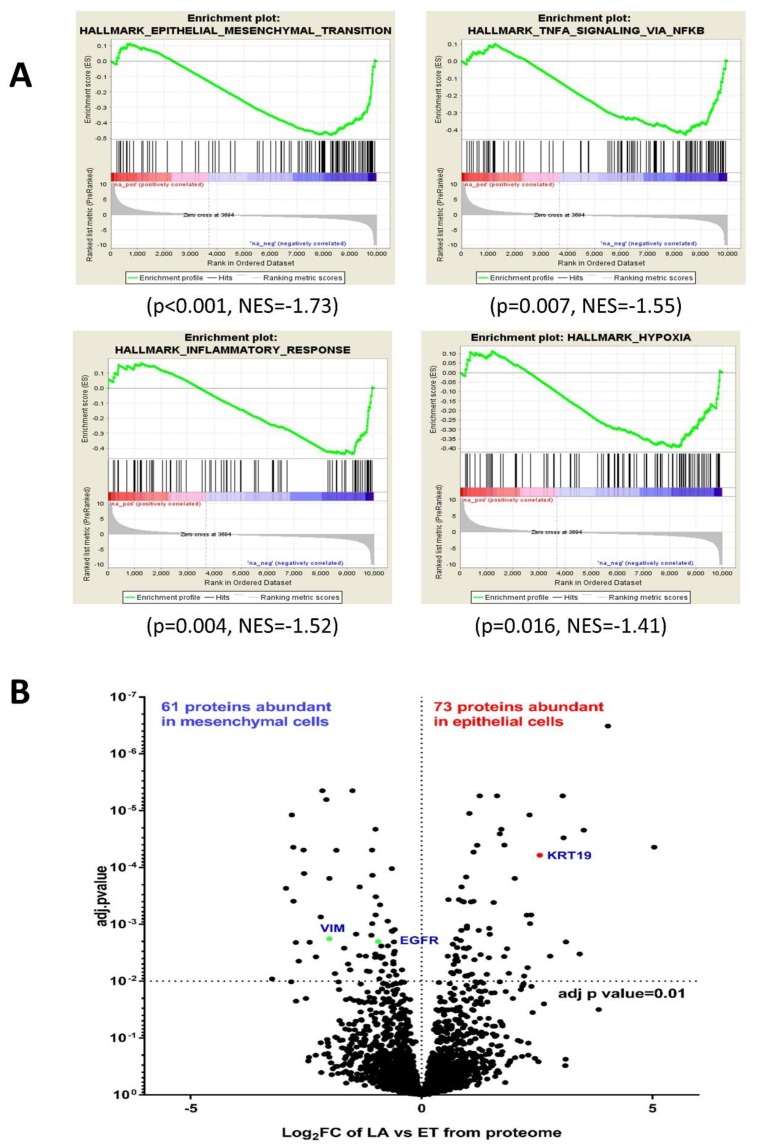
Comparative analysis of transcriptomic and proteomic data from PMC42 cell lines. (**A**) Results from representative enrichment plots from Gene Set Enrichment Analysis (GSEA) (*p <* 0.05) are shown from comparative transcriptome analysis. These data revealed a significant negative enrichment for gene sets involved in EMT in PMC42-LA. (**B**) Quantitative proteome analysis of PMC42-LA in comparison to PMC42-ET reflected in volcano plot shows that 73 proteins were significantly upregulated, and 61 proteins were significantly downregulated.

RNA-seq results obtained previously for the EGF-induced EMT studies in PMC42 system [30] were re-analysed to study the transcriptional differences between the two PMC42 cell lines. Comparative analysis was investigated using the “Hallmark” geneset collection within the MSigDB of GSEA and IPA. Negative enrichment for signatures related to EMT (*p* < 0.001, NES = −1.73), TNFA signalling via NFκb (*p =* 0.007, NES = −1.55), inflammatory response signature (*p =* 0.004, NES = −1.52) and hypoxia signature (*p =* 0.016, NES = −1.41) were observed in PMC42-LA with respect to its parental cell line PMC42-ET using GSEA (Figure 3A). Using IPA for the comparative RNA-seq analysis, the top-five significant upstream regulators we identified were focused on the inhibition of TNF, TGFβ-1, EGFR and JNK gene in PMC42-LA, whereas estrogen receptor gene was considered an activated regulator in PMC42-LA. IPA also reported a significant gene network indicating the importance of TWIST2 downregulation and SPDEF upregulation in the epithelial PMC42-LA cells (Supplementary Figure S2A). 

### 3.4. Comparative Proteome Quantification of Alterations in the PMC42 Cell Line System

We next performed comparative proteomics and subjected protein extracts from the two PMC42 cell lines to mass spectrometry. Among a total of 2460 identified and annotated proteins in the PMC42 cell lines, 244 proteins were expressed at significantly different levels in the two cell lines (adjusted *p* value < 0.01). Of these, 73 proteins were significantly upregulated, and 61 proteins were significantly downregulated by a factor of 2-fold or more in the epithelial PMC42-LA cells. KEGG pathway analysis indicated that differentially regulated proteins were involved in glycolysis/gluconeogenesis (*ALDH1A3, ALDH3A1, ALDOC*) (*p =* 0.00073), proteasome (*PSB3, PSB6, PSB7, PSMD1-4*) (*p =* 6.06 × 10^−8^), protein processing in endoplasmic reticulum (*p =* 3.06 × 10^−6^) and carbon metabolism *(ALDOC, SUCA, G6PD, HXK1, DLDH)* (*p =* 0.00039), respectively. The volcano plot that shows the difference in protein levels between PMC42-ET and PMC42-LA also highlights several EMT markers, among which mesenchymal markers, such as VIM and EGFR, were upregulated by 4-fold (*p* < 2.51 × 10^−5^) and 2-fold (*p* < 5.83 × 10^−5^), respectively, in PMC42-ET cells (Figure 3B). Epithelial markers, such as KRT19 and F11R (Junctional adhesion molecule A), were significantly upregulated in PMC42-LA by 4.75-fold (*p* < 0.00066) and 4.6-fold (*p* < 00093), respectively. IPA also deduced glycolysis (with a z-score of 3.5), aryl hydrocarbon receptor signalling (with a z-score of 3.1) and ILK signalling (with a z-score of 1.6) as significantly upregulated canonical pathways in the epithelial PMC42-LA cell line. Gene network enrichment plot from proteomics analysis indicated a possible role of NFκb complex dysregulation in PMC42-LA cells (Supplementary Figure S2B), which is also in agreement with the similar GSEA findings from RNA-seq analysis. 

### 3.5. Karyotypic Heterogeneity Exists within and across the Sister Breast Cancer Cell Lines PMC42-ET and PMC42-LA

Chromosomal instability (CIN), including numerical CIN (resulting in aneuploidy) and structural CIN (resulting in partial chromosomal gains and losses and translocations) are inherent in cancer and underpin many of the phenotypic manifestations that contribute to cancer progression. Hence, in order to assess their possible contributions to the spontaneous MET seen in the PMC42 system, we explored whether karyotype differences existed in the PMC42 cell lines using metaphase spreads and karyotypic G-banding.

**Figure 4:**
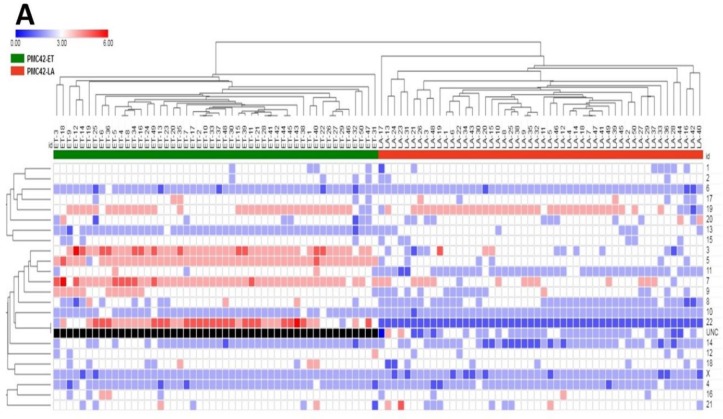

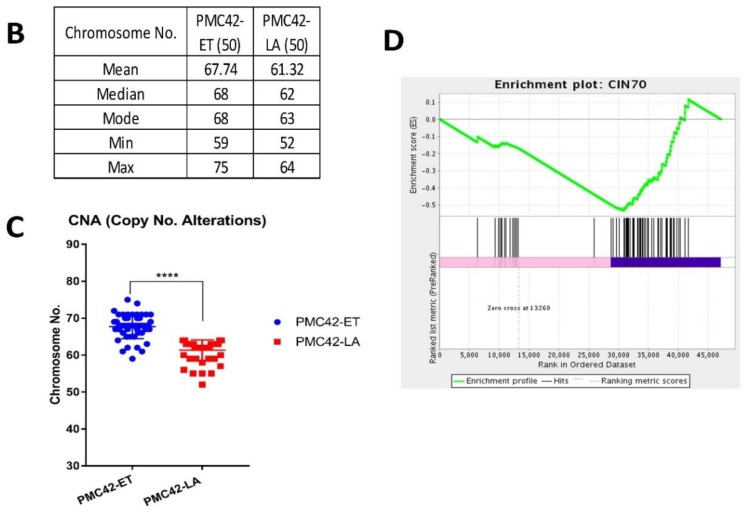
Chromosomal ploidy distribution of PMC42 cell lines. (**A**) Heatmap for copy number distribution of chromosomes deciphered from 50 karyotypes from each of the PMC42-ET and PMC42-LA cell lines *(UNC: Unidentified Chromosome)*. (**B**) Distribution of chromosome numbers of PMC42-ET and PMC42-LA cell lines. (**C**) Chromosome numbers analysed from PMC42-ET and PMC42-LA for a total of 50 cells were compared for each cell line. Significance was determined by an unpaired *t* test with Welch’s correction, with **** *p* < 0.0001. (**D**) CIN70 enrichment plot following Gene Set Enrichment Analysis.

First, we determined the copy number status per chromosome. Chromosomal counts from individual cells in each PMC42 cell line were plotted as a heatmap, where PMC42-ET and PMC42-LA cells clearly are seen as two separate clusters (Figure 4A). CIN, and resulting aneuploidy, is a hallmark of cancer, and despite this strong partition into 2 clusters, variable ploidy distribution was also reflected within each of the PMC42 cell lines. We identified copy number differences of individual chromosomes (Figure 4A). Significant differences in the ploidy levels between the cell lines are represented in Table 1, where eight chromosomes show high chromosomal number differences across the two cell lines. Tetrasomy was observed more often for chromosomes 3, 5, 7, 19 and 22 in PMC42-ET, whereas in PMC42-LA, tetrasomy was observed for chromosomes 7 and 19. Loss of chromosome 22 was found in 100% of PMC42-LA cells, as only a single copy was present in each of 50 cells assessed for karyotyping. The patterns of positive and negative chromosomal correlation with regards to their ploidy were also studied for individual cells in the two PMC42 cell lines, where no strongly significant association was confirmed for PMC42-LA, but positive correlations were observed between chromosomes 9 and 14, and 17 and 20, respectively (*r*^2^ = 0.67 and 0.59, Pearson correlation) (*p*-value of 7.64 × 10^−8^ and 6.37 × 10^−6^), and a negative correlation (*r*^2^ = −0.51) (*p*-value of 0.0001) was found between chromosomes 9 and 12, in the PMC42-ET cell line (Supplementary Tables S3 and S4). Overall, PMC42-ET was primarily comprised of near-triploid karyotypes with a modal number of 68 chromosomes (range 59–75), whereas PMC42-LA was primarily comprised of near-triploid karyotypes with a modal chromosome number of 63 (range 52–64) (Figure 4B). The total chromosome numbers in the two cell lines are significantly different (*p <* 0.0001) (Figure 4C). Two observations suggest an increased level of CIN in PMC42-ET compared to PMC42-LA. First, PMC42-ET cells show a broader range of chromosome numbers, 17, compared to 13 in PMC42-LA cells and more individual chromosome numbers deviate from the modal number (Supplementary Figure S3A,B). Second, GSEA on our RNA-seq data shows a significant depletion in the expression of 70 genes that are part of the well-established CIN70 signature [67] in PMC42-LA cells compared to PMC42-ET cells (*p =* 0.027; Figure 4D). This suggests that CIN may have promoted the transition from PMC42-ET to PMC42-LA.

**Figure 5:**
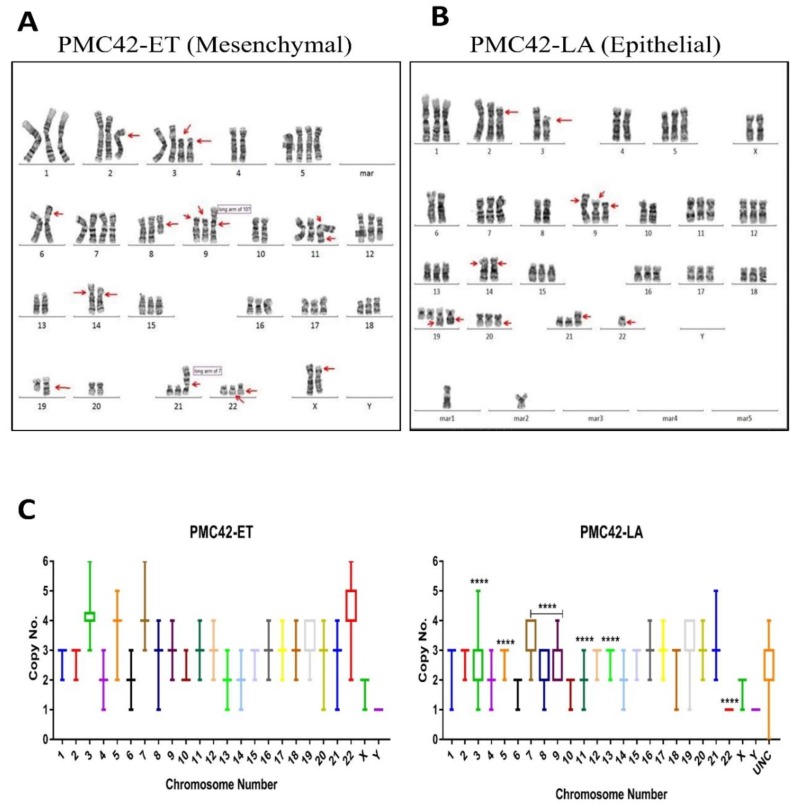
Karyotypic analysis of PMC42 cell lines. (**A**) A representative G-banded karyotype of the near-triploid cell line PMC42-ET, showing structural and numerical changes. (**B**) A representative G-banded karyotype of the cell line PMC42-LA. *Arrows* point to main chromosomal alterations. *Mar_n_* marker chromosome (**C**) Ploidy distribution of each chromosome is presented for PMC42-ET and PMC42-LA from 50 karyotyped cells. *P*-values are indicated (as described in Table 1 using Student's *t*-test), and data presented in box (median, first and third quartiles) and whisker (extreme value) plots *(UNC: Unidentified Chromosome)*.

Next, as expected, karyotyping revealed that the PMC42-LA derivative cell line harbours some of the major structural rearrangements seen in the parental PMC42-ET cells. For example, one of the arms of each of chromosomes 2, 3 and 8 is shorter or truncated, the *p*-arm of chromosome 9 is fused with the long arm of chromosome 10, and chromosome 21 has a third copy of its *p*-arm fused with the long arm of chromosome 7 (Figure 5A,B). Some characteristics, such as truncated arms of chromosomes 2 and 3, trisomy 1 and trisomy 20, and a modal chromosomal number of 66, were consistent with initial reports in 1983 [42]. In PMC42-LA cells only, we also observed a few (in the range of 1–4) marker chromosomes whose derivative chromosomal origins cannot be recognized via karyotyping (Figure 5B, marked as 'mar' and Figure 5C, marked as 'UNC'). Ploidy distributions of each chromosome from 50 karyotyped cells of each cell line also reflect the dynamics of copy number alterations at the chromosomal level (Figure 5C).

Taken together, these results indicate that numerical chromosomal heterogeneity exists between and within the PMC42 cell lines. In addition, PMC42-ET cells show features of CIN and PMC42-LA cells harbour some specific structural abnormalities not observed in PMC42-ET cells. These changes could underpin the transition of PMC42-ET cells to a more epithelial phenotype.

### 3.6. Cancer Driver Mutations in PMC42 Cell Lines

**Figure 6:**
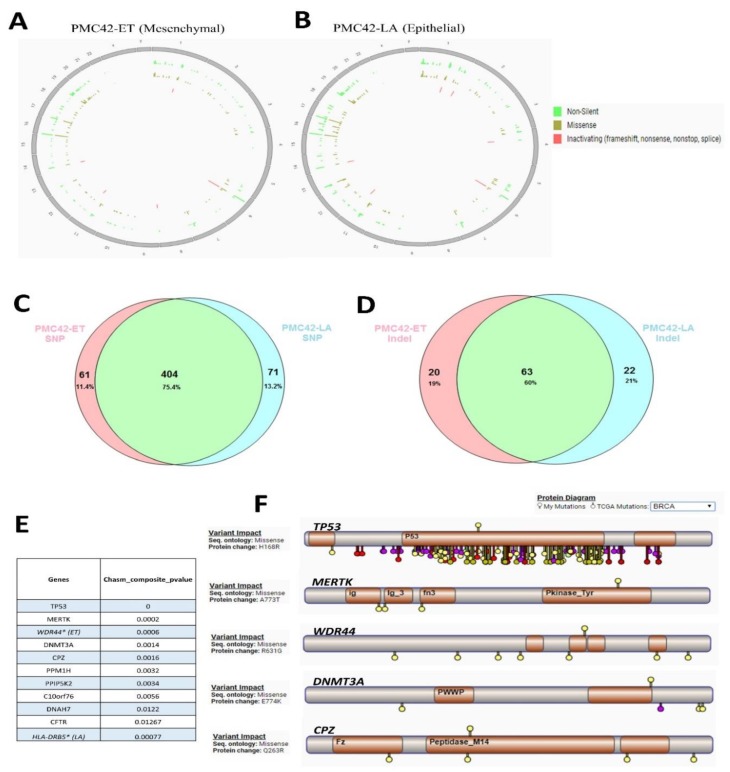
Assessment of whole-exome sequencing (WES) from PMC42 cell lines. Chromosome-specific distribution of non-silent, missense, and inactivating mutations are displayed on Circos plots for PMC42-ET (**A**) and the derivative PMC42-LA (**B**) cell lines, respectively. Representation of shared and unique (**C**) SNVs and (**D**) indels discovered by WES for PMC42-ET and PMC42-LA. (**E**) CHASM score was computed and (**F**) the top 10 potential driver mutations for PMC42-ET and PMC42-LA were determined. The top 5 potential driver mutations for PMC42-ET and PMC42-LA were annotated for their presence within protein sequences (indicated on the top of each gene) and compared for somatic mutations identified in TCGA dataset (indicated on the bottom of each gene) for the same protein sequences, using the software CRAVAT [68].

WES was performed to probe more deeply the genetic aberrations in the PMC42 system. After applying selective filters for delineating deleterious mutations within exons, we identified 465 SNVs in PMC42-ET and 475 SNVs in PMC42-LA (Figure 6A,B). We considered missense, non-sense, frame-shift and splice site mutations that involve structural and functional alteration of the protein products as deleterious. The number of Indels present in PMC42-ET were 83 and in PMC42-LA were 85. Approximately 75.4% of the somatic mutations and 60% of the Indels were shared between the parental and derivative cell line (Figure 6C,D). The results of deleterious SNV and Indels identified using WES across PMC42-ET and PMC42-LA cells are shown in the supplementary document (Supplementary Tables S5 and S6 for PMC42-ET and Tables S7 and S8 for PMC42-LA). CHASM score was computed for all the missense mutations identified in the PMC42 cell lines to identify driver mutations. The top 9 potential drivers that were common between the two PMC42 cell lines were *TP53,* *MERTK, DNMT3A, CPZ, PPM1H, PPIP5K2, C10orf76, DNAH7, CFTR* (Figure 6E). We also compared the driver mutations identified in the PMC42 cell lines with the TCGA mutations dataset using the CRAVAT interface (Figure 6F). The TP53 mutation site (H36R) was reported earlier in the TCGA dataset, whereas the other top 4 driver missense mutation locations identified were considered novel, as they were not reported in the TCGA dataset (as observed from the CRAVAT interface). The deleterious genes identified in the PMC42 cell lines were also stratified in a gene-family matrix according to their known role in cancers using the Broad Institute’s GSEA analysis. We identified three commonly mutated tumour suppressor genes between two PMC42 cell lines (*NF2 (attaining stop gain function), TP53, TSC2)* and an additional mutated tumour suppressor gene *ATM* in PMC42-ET only. Eight commonly mutated oncogenes (*ARNT (TF), EML4, GNAS, NTRK3 (PK), PER1 (TF), TCL1A (TF), TLX1, TTL)* and 2 additional mutated oncogenes *TAL1 and IL6ST* in PMC42-ET only were also identified for their role in tumorigenesis (Table 2; Table 3). Interestingly, the gene-family matrix derived for the deleterious genes in the PMC42-LA cell line in reference to parental PMC42-ET cell line identifies mutations in 2 significant EMT-promoting transcription factors, *SNAI2* (K188N) and *SOX3* (R22P) (Supplementary Tables S9 and S10) and other EMT genes, such as *GSN (R397W), WNT1 (T363K), ITGA4 (R565W) and NID2 (G426E)* (Supplementary Table S7). In addition to that, the EMT-associated splice variation regulator *ESRP2* (R248S) was found to be mutated in the parental PMC42-ET cell line.

### 3.7. Inference of CNV from Exome Sequencing Data

**Figure 7:**
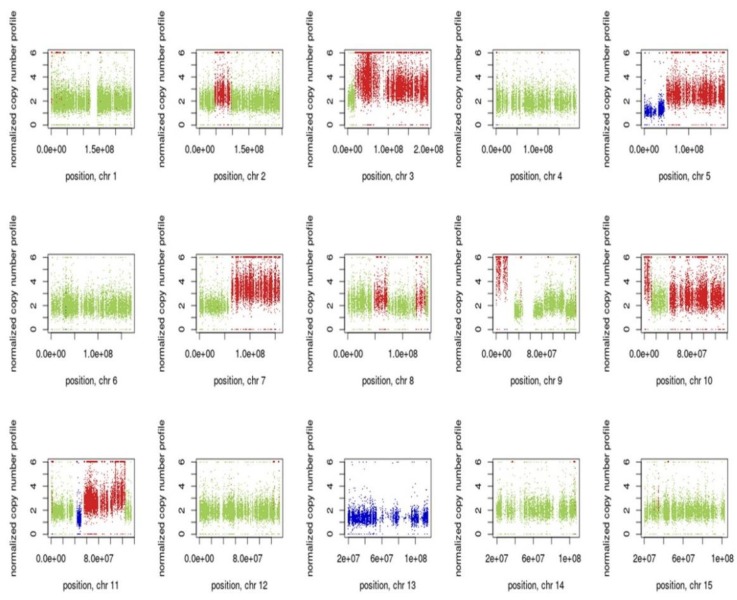

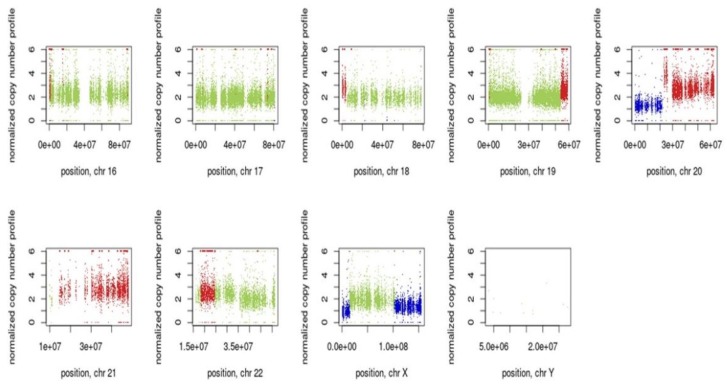
Visualization of Control-FREEC v 6.0 output from PMC42 cell lines exome sequencing data (Illumina HiSeq 2000). Copy number profiles for all chromosomes are shown of PMC42-ET in comparison to PMC42-LA, normal copy number status is shown in green, copy number gains are reflected in red and copy number losses are shown in blue.

The estimated copy number ratios of chromosomal segments for PMC42-ET relative to PMC42-LA are shown in Figure 7. Control-FREEC was used to determine the copy number ratio profiles and to identify regions with significant amplification or loss. The copy number profiles deduced from WES resulted in a total of 166 gain and 34 loss segments in PMC42-ET, relative to PMC42-LA. The most significant losses are from 5p, 20p, Xq and whole chromosome 13, while the major gains are in chromosomes 3, 5q, 7q, 10q, 11q, 20q and 21. The identified genomic regions of amplification or loss were also consistent with our karyotyping studies. Copy number changes of the regions (i.e., amplification of chromosomes 3, 5, 7, 9,11, 22 and loss of chromosomes 13 in PMC42-ET relative to PMC42-LA) detected in the WES study were also identified in karyotyping. Additionally, WES data reflects amplification in chromosomes 10q and 21, which might be due to nonreciprocal translocation events of an additional chromosome 9 with chromosome 10q and chromosome 21 with 7q as shown in karyotype analysis. WES helps in revealing the amplifications/losses at the gene level, whereas karyotyping reflects the overall ploidy distribution better (as reflected in Figure 5), as it is a representation of single cells. The amplified/lost regions from WES data for PMC42-ET with respect to PMC42-LA is also provided in additional Supplementary Table S11.

### 3.8. TGFBR2 Ablation and Influence on EMT Induction in PMC42-LA

**Figure 8:**
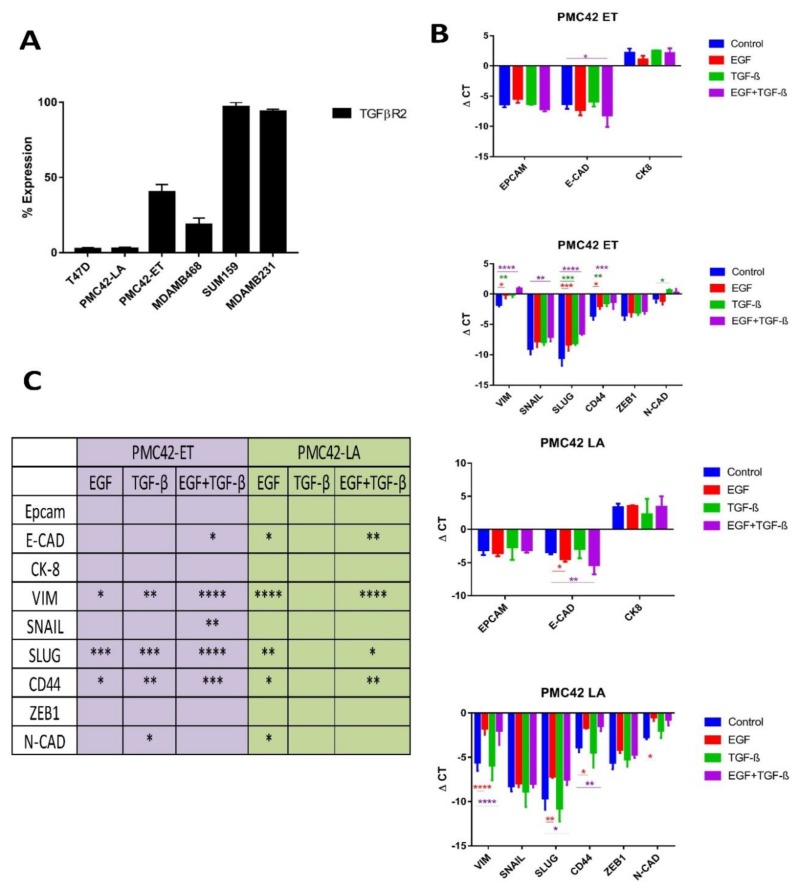
TGFBR2 ablation and influence on EMT induction in PMC42-LA. (**A**) Cell surface expression levels of TGFβR2 in a panel of breast cancer cell lines representative of distinct molecular subtypes. (**B**) PMC42-ET and PMC42-LA cells were treated for 5 days with 10 ng/mL EGF, 10 ng/mL TGF-β and combined 10 ng/mL of EGF and TGF-β. qPCR analysis of epithelial and mesenchymal markers were tested after EMT induction for 6 days with growth factor treatments. dCt values normalized against L32 and as an average from triplicates are shown. Statistical method applied is a two-way ANOVA with * indicating a *p*-value < 0.1, ** *p*-value < 0.01, *** *p*-value < 0.001 and **** *p*-value < 0.0001. (**C**) *p*-values calculated using 2-way ANOVA against each gene expression are tabulated.

When analyzing the segments of genes that were completely lost in either of the PMC42 cell lines, we identified two regions from PMC42-LA that were completely missing. The chromosome 1 region carrying genes *FOXD2, FOXE3* and the chromosome 3 region containing genes *GADL1, RBMS3, TGFBR2* were completely lost from the PMC42-LA cell line. The specific functions of *FOXD2* and *FOXE3* genes are yet to be determined, however the role of *TGFBR2* in EMT induction is well established [27,69,70]. The surface expression of TGFβR2 in the PMC42 system was analysed in comparison with several other cell lines (T47D, MDA-MB-468, SUM-159 and MDA-MB-231) using FACS (Figure 8A). High TGFβR2 surface expression was seen in almost all the cells for Basal B cell lines SUM-159 and MBA-MB-231, whereas only 40% of PMC42-ET cells expressed TGFβR2 on their surface, and TGFβR2 expression was completely absent on PMC42-LA cells, which is in concordance with our WES-deduced results. The surface expression of TGFBR2 on T47D cells was also negligible, consistent with their low EMT-associated TGF-β enrichment score (TES) [27]. RNA-seq analysis of the PMC42 cell lines was also interrogated using the algorithms as described [27] to obtain their TES values and identify any evidence of intrinsic TGF-β-induced EMT. The PMC42-ET had a TES value of 0.594 which is relatively high compared to the PMC42-LA TES value of −0.015. The low/negative TES value of PMC42-LA is also in concordance with the previously deduced TES values from Luminal and Basal A cell lines (MCF7: −0.58977; T47D: −0.69277; MDAMB468: −0.66892), whereas Basal B cell lines have relatively higher TES values (MDA-MB-231 0.130999; SUM159PT: 0.430126). TES values for the various breast cancer cell lines are taken from Supplementary data file S10 of [27]. 

PMC42-ET and PMC42-LA cell lines were also tested for their EMT induction with EGF, TGF-β and combined treatments using RT-qPCR (Figure 8B). E-cadherin was significantly downregulated with combined EGF and TGF-β growth factor treatments in the PMC42-ET cell line compared to either EGF or TGFβ alone, suggesting that TGF-β augments the previously reported EGF-induced EMT [30]. There was significant upregulation of mesenchymal markers vimentin, Slug and CD44 in both the cell lines with EGF, and with combined EGF and TGF-β treatment in PMC42-ET cells, however there were no effects of TGF-β treatments on PMC42-LA cells (Figure 8B,C). At an individual factor level, assessed via transcriptomics, several modulators or mediators of TGF-β signalling were impacted across PMC42 cell lines (e.g., AGR2, RhoA, TGFB1, CTNNB1, JUNB) were significantly downregulated in PMC42-LA. Thus, the PMC42-LA cell line did not display any predisposition to undergo EMT-like changes in mesenchymal gene expression with TGF-β treatment.

### 3.9. Inter-Data Relationships from CNV and RNA-seq with Proteome Data

**Figure 9:**
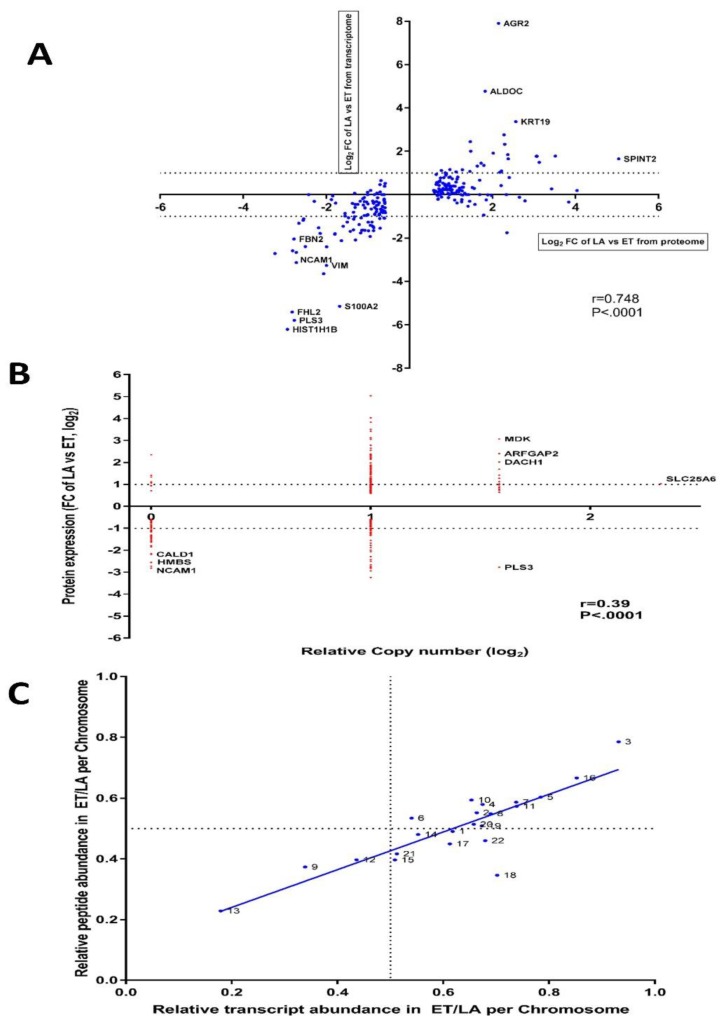
Inter-data relationships from CNV, RNA-seq and proteome studies in PMC42 cell lines. (**A**) Log_2_ fold change of mRNA and protein expression levels of 244 significantly differential expressed proteins for PMC42-LA vs PMC42 ET were computed. Spearman’s correlation coefficient (*r* with *p*-value) between log_2_ fold change of protein and mRNA expression is indicated at the bottom right. Dotted horizontal bars indicate 2-fold upregulation and downregulation on the log_2_ scale for mRNA expression. (**B**) Log_2_ fold change of 244 significantly differential expressed proteins were linked to the genomic copy number and spearman’s correlation coefficient (*r* with *p*-value) is indicated at the bottom right. (**C**) Correlation between relative peptide and transcriptome abundance in PMC42-ET vs. PMC42-LA per genomic coordinate. Correlation analysis was performed in GraphPad Prism with *R*^2^ value of 0.7361 (*p* < 0.0001).

Correlations were assessed between protein expression and gene expression, and between protein expression and copy number variation, after applying the filter to only those proteins (*n* = 244) that were significantly dysregulated. For the RNAseq-to-proteome comparison, the Spearman correlation coefficient was 0.748 (Figure 9A), whereas the correlation was only 0.39 for CNV deciphered from WES at the gene level compared to the proteome (Figure 9B). Assessment of impact of CNV changes at whole differential RNA expression level reflects a Spearman’s correlation coefficient of *r* = 0.002805, with a non-significant *p* value of 0.77 (Supplementary Figure S4). Undoubtedly, some of the gene expression levels are influenced by changes in amplification or depletion of gene dosage at allele level, but this is probably masked by the likelihood that gene expression can be significantly modulated by other factors, such as epigenetics or transcription factors. Interestingly, when extrapolating RNA-seq and proteome results for the 244 differential expressed proteins, AGR2 shows relatively very high gene expression in PMC42-LA compared to PMC42-ET. An essential role of induced AGR2 in re-acquisition of epithelial markers has been reported [71], where activated Smad and Erk signalling cascades were identified as mutually complementary pathways responsible for TGF-β-mediated inhibition of AGR2. Since TGFBR2 expression is absent in PMC42-LA, AGR2 may be playing a crucial role in maintaining the epithelial phenotype of PMC42-LA cells. This is consistent with a strong enrichment of ARG2 expression in the Luminal subgroup of breast cancer cell lines, and some enrichment in Basal A, compared to Basal B (Supplementary Figure S3C). 

### 3.10. The Differences in PMC42 Karyotypes are Reflected in Their Transcriptome and Proteome Ratios

The significant genomic differences in the PMC42 cell lines led us to ask whether the changes in chromosome content mediated changes in transcriptome and proteome that could influence phenotype determination. The relative transcriptome and proteome abundance for a given chromosome across the two PMC42 cell lines were computed from log_2_-transformed fold changes of each transcript and proteome. To reduce the noise from the transcriptomic abundance, transcripts that were not expressed or for which normalized values were less than 10 in both the cell lines were discarded. The results show remarkable concordance between the chromosome copy number content and the corresponding transcript and protein abundance from chromosome 3 and chromosome 13 (Figure 9C). PMC42-ET cells have four copies of chromosome 3 on average, whereas PMC42-LA cells have an average of 2 copies, while PMC42-LA cells have three copies of chromosome 13 on average, whereas two copies are present in PMC42-ET. Apart for chromosome 3, transcript and proteome abundance from chromosomes 5, 7, 10 and 16 is also higher in PMC42-ET relative to PMC42-LA, which also corresponds to their higher ploidy distribution in PMC42-ET, except for chromosome 16. Strikingly, the discriminant gene analysis performed for chromosomes 13 and 3 also identified specific genes on chromosome 13 (*DNAJC15, SPG20, SLITRK6* and *DACH1*) that had significant overlap in GSEA with genes down-regulated in TMX2-28 cells (breast cancer) which do not express ESR1 [Gene ID = 2099] compared to the parental MCF-7 cells, which do [72], (*p*-value 1.6 × 10^−5^). Therefore, we hypothesised that the gain in chromosome 13 upregulated the expression of various genes that drives the signalling mechanism of ER in PMC42-LA, causing it to be represented as an upstream regulator in our comparative IPA findings. The results show a high degree of concordance between the relative transcript and proteome abundance across the PMC42 cell lines (*r*^2^ = 0.736). 

### 3.11. Bioenergetic Profiles of PMC42 Cells in Comparison with Other Breast Cancer Cell Lines

**Figure 10:**
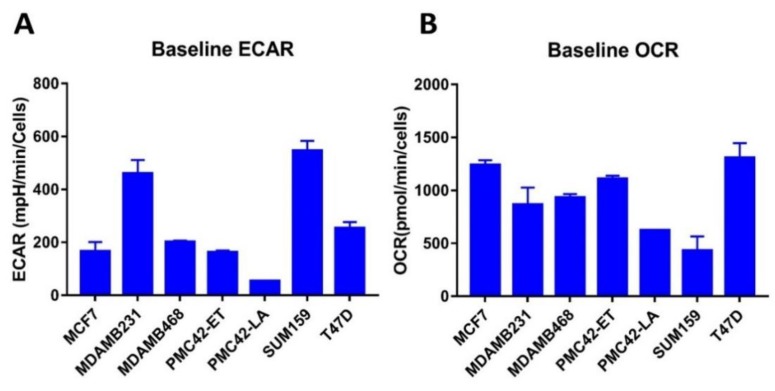

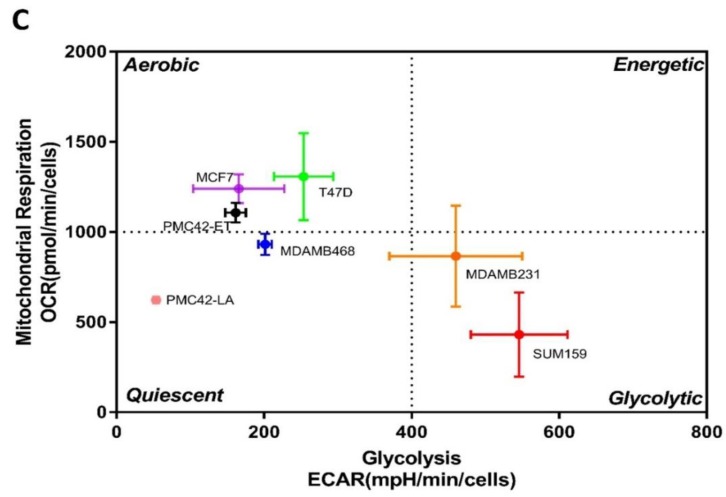
Metabolic profile of PMC42-ET, PMC42-LA and a panel of breast cancer cell lines representative of distinct molecular subtypes. (**A**) Extracellular acidification rate (ECAR) and (**B**) Basal oxygen consumption rate (OCR) measurements. (**C**) OCR: ECAR quadrant showing the bioenergetics phenotype of cell lines using Seahorse analyser (data presented as mean ± s.d., *n* = 3).

Since glycolysis was one of the significantly attenuated pathways identified in proteome analysis, we also evaluated the mitochondrial bioenergetic profiles of PMC42 cell lines by measuring their oxygen consumption and glycolysis rates in comparison with the other four breast cancer cell lines: MCF-7, T47D (Luminal), MDA-MB-468 (Basal A) and MDA-MB-231, SUM-159 (Basal B). The extracellular acidification rate (ECAR) and oxygen consumption rate (OCR), as indicators of lactic acid production during glycolysis and mitochondrial respiration during OXPHOS, respectively, were measured (Figure 10). Interestingly, the baseline ECAR status of PMC42-LA was lower than all other cell lines evaluated (Figure 10A). Basal B/mesenchymal MDA-MB-231 and SUM-159 cell lines exhibited higher ECAR as compared to all other cell lines. The higher OCR was seen in luminal MCF-7 and T47D cell lines (Figure 10B). 

## 4. Discussion

**Figure 11:**
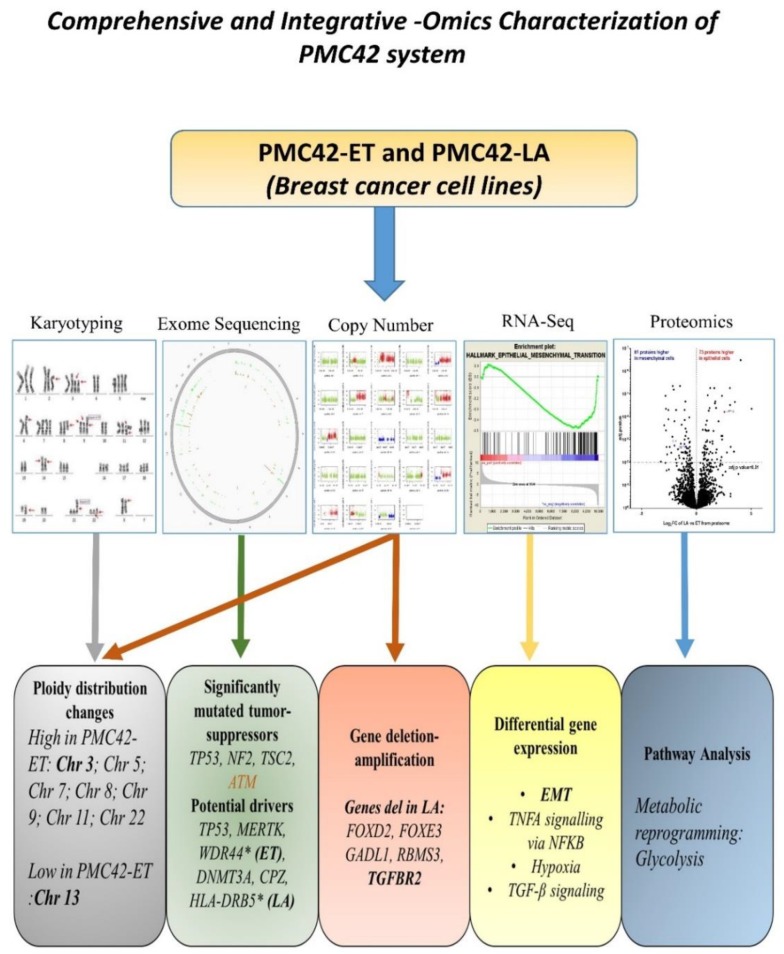
Graphical abstract reflecting the multiple outcomes from the comprehensive and integrative—omics characterization and karyotyping of the PMC42 model system.

Although the requirement of MET in metastasis is somewhat controversial [21,73,74,75], the transition of mesenchymally orientated cancer cells to a more epithelial state has been shown to allow cancer cells to survive and seed in distant sites prior to development of a metastatic lesion [18,68,76,77,78]. The comprehensive integrated analysis of the PMC42 system enhances our understanding of the regulation of molecular events relevant to MET change in the context of breast cancer. In this study, utilization of several omics (exome, transcriptome, proteome) platforms, along with karyotyping and metabolic status, has allowed integrative insights not possible with isolated studies (Figure 11). The PMC42 cell line model system comprises a mesenchymal, parental PMC42-ET cell line and an epithelial derivative PMC42-LA cell line that exhibits profound morphological changes [40], decreased cellular proliferation, distinct karyotype, depletion of TGFBR2 gene, distinct pathways mediated by TNF-alpha signalling, and decreased metabolic bioenergetics. 

Our study identified common canonical driver mutations in TP53, MERTK and DNMT3A in the PMC42 system, as well as a number of unique molecular alterations in the PMC42-ET and PMC42-LA genes that are mutated (Supplementary Tables S5–S8) and have thus potentially impacted the phenotypic heterogeneity seen. Some of the allelic heterogeneous mutations detected in PMC42-LA have also been associated with EMT drivers or markers; we identified novel mutations in *GSN (R397W), WNT1 (T363K), SNAI2 (K188N), ITGA4 (R565W), SOX3 (R22P) and NID2 (G426E)* (Supplementary Table S7). SNAI2-null mice are reported to have retarded epithelial migration rates [79], and SOX3 has been implicated in the malignant behaviour of glioblastoma [80], EMT, and in the promotion of migration and invasion of osteosarcoma cells [81,82]. A N161K mutation in SOX3 was found associated with progression of SCLC along with other mutated SOX members [83]. The implications of genomic variants for these other markers have not been studied or reported in context of EMT. These identified mutations in EMT drivers evaluated at differential gene expression level reflected complete absence of expression of WNT1 and 6-fold downregulation of ITGA4 in PMC42-LA cells.

Comparison of transcriptome and proteome analyses across PMC42 cell lines, combined with GSEA and IPA, were performed to gain insights into the biological processes dysregulated within our model system. In our transcriptome studies, the expression of forkhead box A1 (FOXA1) gene, which acts to control transcription of estrogen receptor-regulated genes and repress the basallike features of breast cancer cells [84,85,86,87], was observed only in PMC42-LA. We observed upregulation in the expression of several specific genes that favour the MET in PMC42-LA, including AGR2 and GRHL2. The most prominent feature of GSEA analysis was the strong down-regulation of EMT in PMC42-LA (*p*-value of < 0.001) (Figure 3). These results indicate that this model system is highly appropriate for studies of EMP [40]. Interestingly, the combined analysis of both transcriptome and proteome across PMC42 cell lines using IPA also indicated that EGFR and ER may be common upstream regulators that are dysregulated between the two cell lines. The ability of EGFR signalling to drive EMT is widely reported in literature [88,89,90] and is also considered an important driver of MDA-MB-231 invasion leading to formation of brain metastasis [91]. Studies from GSEA and IPA also highlighted an involvement of the TNF-α pathway, mediated by NFKB (*p*-value 0.007), as a major attenuated pathway between PMC42 cell lines. Significant assessment from in vitro studies have highlighted the significance of various cytokines, such as TNF-α [92,93] and growth factors such as EGF and TGF-β in mediating EMT changes, and how these targets can have therapeutic implications in combating EMP [20,93,94]. 

Furthermore, our recent published work also investigated the dynamic interconversions observed between the transitional epithelial and mesenchymal subpopulations delineated by EpCAM profiling in the predominantly epithelial PMC42-LA breast cancer cells. The subtleties of this transition vary in proportion of epithelial and mesenchymal phenotypes as determined from single cells clonal propagation. Differences observed in the functional attributes of the single cell-derived clones further explains the stochastic nature and the intrinsic cellular plasticity in PMC42-LA. Interestingly, the implementation of whole exome sequencing across the EpCAM-high and -low subpopulation indicates that observed intrinsic phenotypic plasticity in PMC42-LA was not attributable to chromosomal instability [66]. Moreover, the PMC42-LA subline derived from PMC42-ET and remaining phenotypically stable over two decades has maintained distinct karyotype with reduced number of chromosomes and significant ploidy disparity reflected for eight of the chromosomes.

In this pattern of karyotypic differences reflected in the genomic copy number alterations across the two cell lines, we observed that the allele fractions had significantly deviated in the PMC42-LA daughter cell line (*p*-value < 0.0001). PMC42-ET has the higher chromosome number, consistent with a relative increase of its transcriptome and proteome abundance (Figure 9C), a broader range of chromosome numbers, and a higher CIN70 score (Figure 4 and Figure 5). The gain of chromosome 13 and the loss of chromosome 3 in PMC42-LA were the most prominent changes reflected in both the transcriptome and proteome (Figure 9C). Strikingly, the locus of TGFBR2 gene is also on chromosome 3. Notably, we observe a potential link between the deletion affecting the TGF-β receptor gene TGFBR2 in PMC42-LA and its negative TES scores assessed from transcriptome analysis. Moreover, the aberration of TGFBR2 also made PMC42-LA cell line non-responsive to TGF-β1 stimulus for EMT mediated changes (Figure 8C). Thus, changes in specific chromosomes in PMC42-ET, with the acquisition of new mutations and genetic deletion of TGFBR2, may have contributed to the derivation of the MET observed in the PMC42-LA cells. The assessment of the impact of copy number variations at single-gene level across the whole differential mRNA analysis did not yield a significant association (Supplementary Figure S4). Differential gene expression instead of solely based on amplification of gene copies can also be modulated by other factors, such as gene epigenetics or transcription factors. Indeed, Ohshima et al. had associated the gene expression level of various oncogenes with copy number and found that the R value varied between 0.06 and 0.53 across different cancer datasets [95]. 

CD44 and CD24 are considered putative stem cell markers [96,97] and expression profiling of selected breast cancer cell lines in this study correlates well with previously performed studies on the same cell lines [64,98,99]. Expression of CD24 was equally low or negligible in both the PMC42 cell lines and interestingly, we find higher expression of CD44 in PMC42-LA than anticipated. Many studies have confirmed the CD44^high^/CD24^low^ profile as a reflection of a mesenchymal state [25,39,100]. However, the epithelially shifted PMC42-LA reflected the increase in CD44^high^/CD24^low^ profile as compared to the parental PMC42-ET cells. There are other reports of ambiguity regarding functional aspects of these markers [101], suggesting that further investigations of stemness and the regulatory factors in this cell system are required. 

Understanding the mechanistic basis of metabolic alterations and their role in tumorigenesis is currently an area of intense research interest [102,103,104,105,106]. Despite glycolysis being one of the significantly attenuated pathways in PMC42-LA cells identified through proteomics analysis, Seahorse experiments indicated decreases in both oxidative metabolism and glycolysis in PMC42-LA cells relative to PMC42-ET. This suggests that the observed metabolic alterations may be attributable to differences in mitochondrial number, rather than proteome differences in metabolic genes required for glycolysis: *ALDH1A3*, *ALDH3A1* and *ALDOC* between cell lines. The analysis of metabolic phenotypes emphasises the importance of using bioenergetics profiles to decipher phenotype assessment, rather than drawing conclusions solely from mutational profiling, RNA-seq and/or proteome data [107,108].

## 5. Conclusions

In conclusion, the data we have presented herein underscore profound differences in the PMC42 system by allowing for a comprehensive integration of whole exome sequencing, RNA-seq, proteomics and karyotyping data. The identification of novel somatic mutations and indels provide insights into the differences between the two cell lines at genetic scale that potentially drive the phenotypic differences from the genomic level. The loss of *TGFBR2* gene from the derivative PMC42-LA cell line contributes to a reduction in intrinsic TGFB signalling, and these cells are also refractory to TGF-β-induced EMT, however this pathway did not emerge from GSEA analysis. Comparative RNA-seq also demonstrated the PMC42 system to be an authentic MET model. The inter-data relationships illustrate a high degree of concordance between the relative transcript and proteome abundance and chromosome copy number variations across the PMC42 cell lines and identified putative targets to reverse MET. The novel findings provide mechanistic insights into how genomic instability and karyotypic variations have led to acquisition of new autonomous clonal karyotype and phenotype, which is still related to, but distinct from the parental cell line. However, further metabolomics and epigenetics studies might be more compelling to add into the paradigm to determine implications for mesenchymal to epithelial plasticity changes. Overall, this investigation provides an example of the heterogenous changes that may occur in expanding cancer populations and provides evidence for the levels on which these changes occur to affect their phenotypic properties.

## Figures and Tables

**Table 1:** Ploidy alterations of 50 single cells from PMC42-ET and PMC42-LA were compared using *t*-test.

Chromosome No.	*p*-Value across ET vs. LA
22	5.28847 × 10^−45^
5	1.50398 × 10^−28^
13	7.71473 × 10^−23^
11	9.42164 × 10^−20^
3	3.33503 × 10^−18^
7	8.49205 × 10^−14^
8	3.73987 × 10^−8^
9	1.08547 × 10^−6^
10	0.012162341
X	0.0151172
14	0.022502942
18	0.083804992
12	0.260603283
1	0.278286015
15	0.531258862
2	0.678929758
6	0.717332498
16	0.748135128
4	0.75812698
17	0.771687988
20	0.814301536
21	0.823855275
19	0.928168854

**Table 2:** Gene set enrichment analysis (GSEA) for genes with deleterious mutations (SNV and Indels) identified in PMC42-ET.

	Cytokines and Growth Factors (CGF)	Transcription Factor (TF)	Homeodomain Proteins (HP)	Cell Differentiation Markers (CM)	Protein Kinases (PK)	Translocated Cancer Genes (TCG)	Oncogenes	Tumour Suppressors
**Tumour suppressors**	0	1	0	0	1	0	0	4*
**Oncogenes**	1	4	1	1	1	8	10^&^	
**Translocated cancer genes**	0	4	1	0	1	8*		
**Protein kinases**	0	0	0	0	17^#^			
**Cell differentiation markers**	2	0	0	8^!^				
**Homeodomain proteins**	0	4	4^@^					
**Transcription factor**	0	28^$^						
**Cytokines and Growth Factors**	6^							

Note: (4* tumour suppressor genes are ATM (PK), NF2, TP53 (TF), TSC2 8* **translocated cancer genes/oncogenes** are ARNT (TF), EML4, NTRK3 (PK), PER1 (TF), TAL1 (TF), TCL1A (TF), TLX1, TTL and 2^&^ additional oncogenes are GNAS, IL6ST (CM and CGF) **17^#^ protein kinases** are ANKK1, ATM, AURKB, CSNK1G2, FRK, LRRK2, MAP3K9, MAST4, MERTK, NRBP2, NTRK3, PASK, PKMYT1, PRKDC, ROCK2, SLK, TTN **8^!^ cell differentiation markers** are CR1, CR2, FCGR3A, IGLL1, IL6ST, LILRB5, MSR1, SEMA7A **4^@^ homeodomain proteins** are HOMEZ, IRX3, TLX1, ZEB2 **28^$^ transcription factors** are ARNT, CEBPZ, CHD4, E2F1, ESR1, EYA3, FOXI1, HOMEZ, IRX3, MED21, NEUROD4, NR1I2, PER1, PRDM2, RFX5, RREB1, RRN3, SP1, SRA1, SUPT5H, TAL1, TFDP3, TLX1, TP53, UHRF1, ZEB2, ZNF160, ZNF91 **6^ cytokines and growth factors** are C5, CMTM2, CYR61, IL6ST, SEMA6D, SEMA7A) (Green colour denotes mutations unique to this cell line).

**Table 3:** Gene set enrichment analysis (GSEA) for genes with deleterious mutation (SNV and Indels) identified in PMC42-LA.

	Cytokines and Growth Factors	Transcription Factor	Homeodomain Proteins	Cell Differentiation Markers	Protein Kinases	Translocated Cancer Genes	Oncogenes	Tumour Suppressors
**Tumour suppressors**	0	1	0	0	0	0	0	3*
**Oncogenes**	0	3	1	0	1	7	8^&^	
**Translocated cancer genes**	0	3	1	0	1	7*		
**Protein kinases**	0	0	0	0	15^#^			
**Cell differentiation markers**	1	0	0	6^!^				
**Homeodomain proteins**	0	3	3^@^					
**Transcription factor**	0	24^$^						
**Cytokines and Growth Factors**	5^							

Note: (3* tumour suppressor genes are NF2, TP53 (TF), TSC2 7* **translocated cancer genes/oncogenes** are ARNT (TF), EML4, NTRK3 (PK), PER1 (TF), TCL1A (TF), TLX1, TTL and 1^&^ additional oncogene is GNAS, **15^#^ protein kinases** are ANKK1, AURKB, CSNK1G2, FRK, MAP3K9, MAST4, MERTK, NRBP2, NTRK3, PASK, PKMYT1, RIPK2, ROCK2, RPS6KA5, STK16 **6^!^ cell differentiation markers** are CR1, CR2, FCGR3A, IGLL1, LILRB5, SEMA7A **3^@^ homeodomain proteins** are HOMEZ, TLX1, ZEB2 **24^$^ transcription factors** are ARNT, CEBPZ, CHD4, E2F1, ESR1, FOXI1, HOMEZ, MED21, NEUROD4, NR1I2, PER1, PRDM2, RFX5, RREB1, RRN3, SP1, SRA1, SUPT5H, TLX1, TP53, UHRF1, ZEB2, ZNF160, ZNF318 **5^ cytokines and growth factors** are C5, CMTM2, CYR61, SEMA6D, SEMA7A (Green color denotes mutations unique to this cell line).

## Supplementary Materials

The following are available online at https://www.mdpi.com/2077-0383/8/8/1253/s1.
