# Supplementary material for: Multi-Omics Characterization of the Spontaneous Mesenchymal–Epithelial Transition in the PMC42 Breast Cancer Cell Lines
Source: J Clin Med. 2019 Aug 19;8(8):1253. doi: 10.3390/jcm8081253 (PMC6723942; doi:10.3390/jcm8081253)
Supplement: Supplementary file 1 [file jcm-08-01253-s001.zip › Supplementary Info/Supplementary Figures.pdf]

Journal of
Clinical Medicine

Supplementary Figure 1

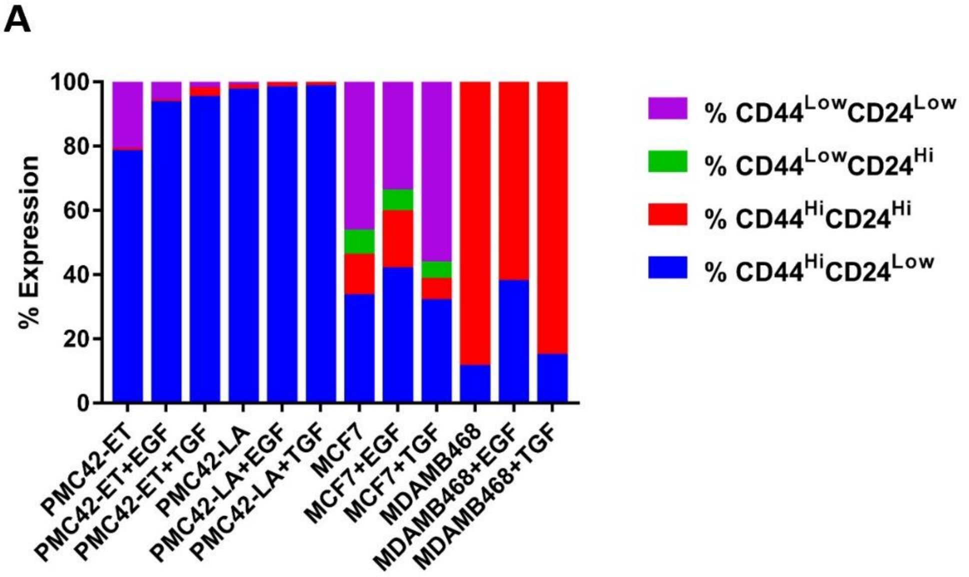

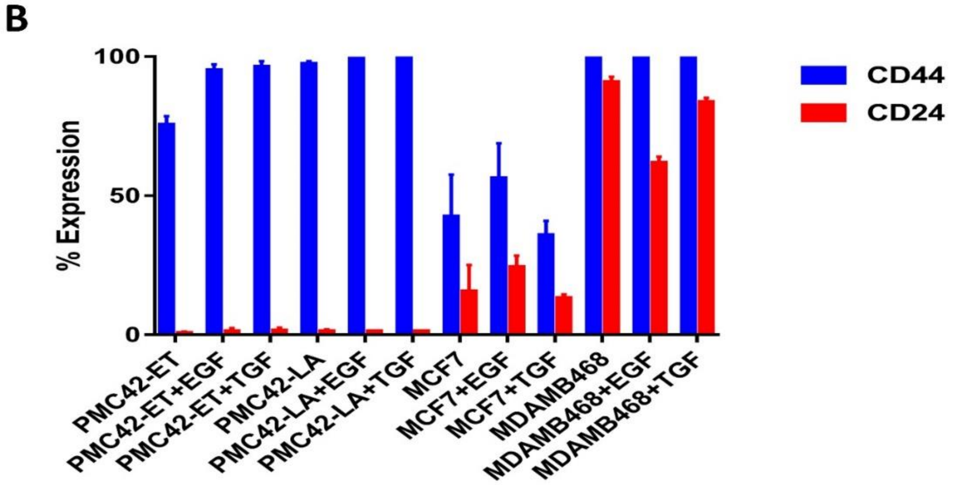

Supplementary Figure S1. Assessment of stem cell markers on BC cell lines with EGF and TGF-β treatments. (**A**) Percentages of the subpopulations defined by the combination of the stem cell markers CD44 and CD24 in a panel of breast cancer cell lines, treated with EGF and TGF-β (10 ng/mL) for 48 h. (**B**) The expression of the stem cell markers CD44-FITC and CD24-PE in a panel of breast cancer cell lines were compared by flow cytometry. Indicated plot is the mean ± SD of three independent experiments.

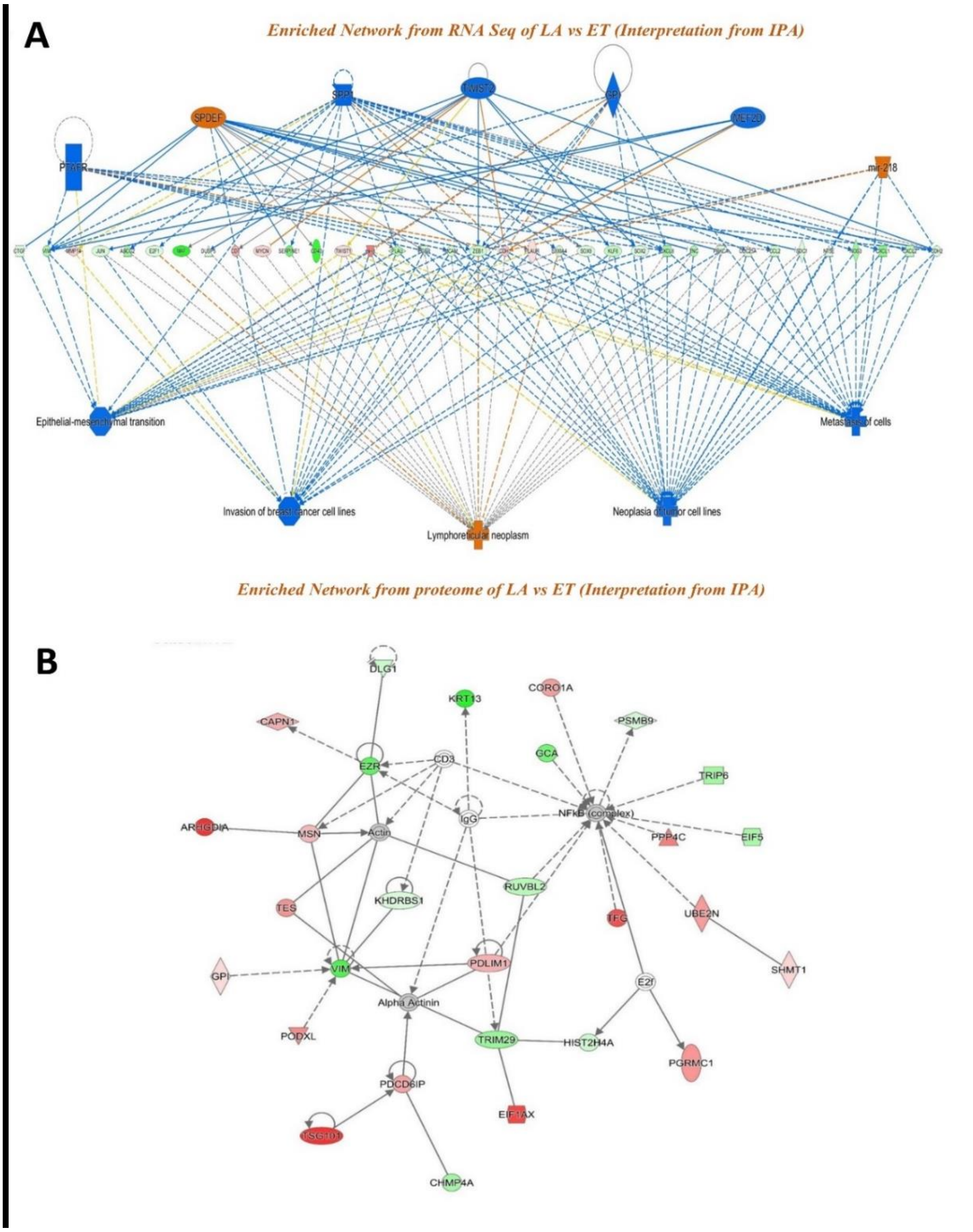

Supplementary Figure S2. Enriched network plots deduced from Ingenuity Pathway Analysis® (IPA). Top enriched network identified from the IPA assessment for comparative transcriptome (**A**) and proteome (**B**) analysis of PMC42 cell lines. The edges are coloured orange when leading to activation of the downstream node, blue when leading to its inhibition, and yellow if the findings underlying the relationship are inconsistent with the state of the downstream node.

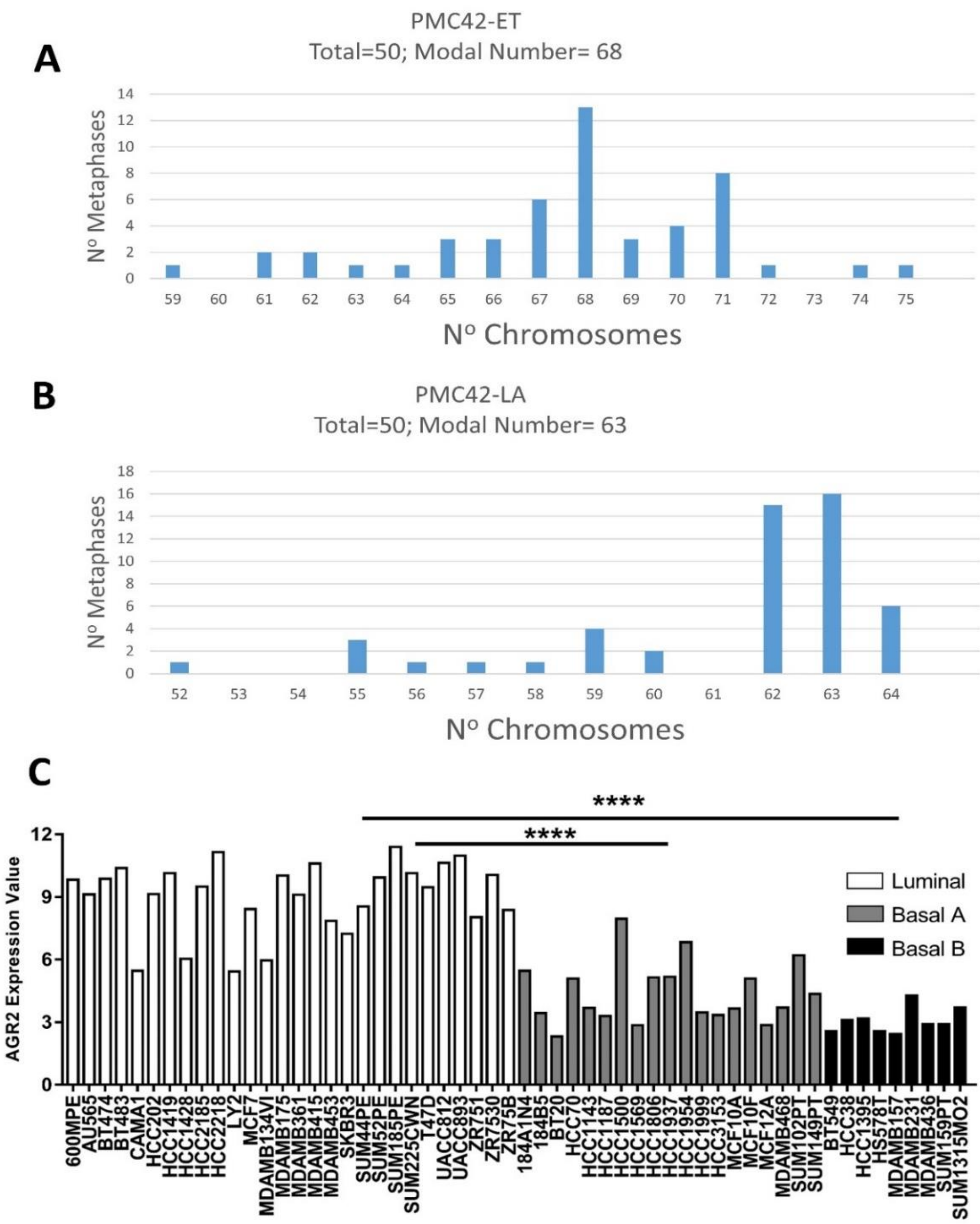

Supplementary Figure S3. Distribution of chromosome numbers of PMC42-ET (**A**) and PMC42-LA (**B**) cell lines. A total of 50 metaphases were analysed in PMC42-LA and PMC42-ET, respectively. (**C**) ARG2 assessment in gene expression data of 50 breast cancer cell lines and five non-malignant breast cell lines, including three subtypes of luminal, basal A and basal B/mesenchymal. Data are from Array Express (accession no. E-MTAB-181) (Heiser et al., 2012) and are normalized log_2-transformed values; **** $p < 0.0001$ (one-way ANOVA, with Tukey's multiple comparisons).

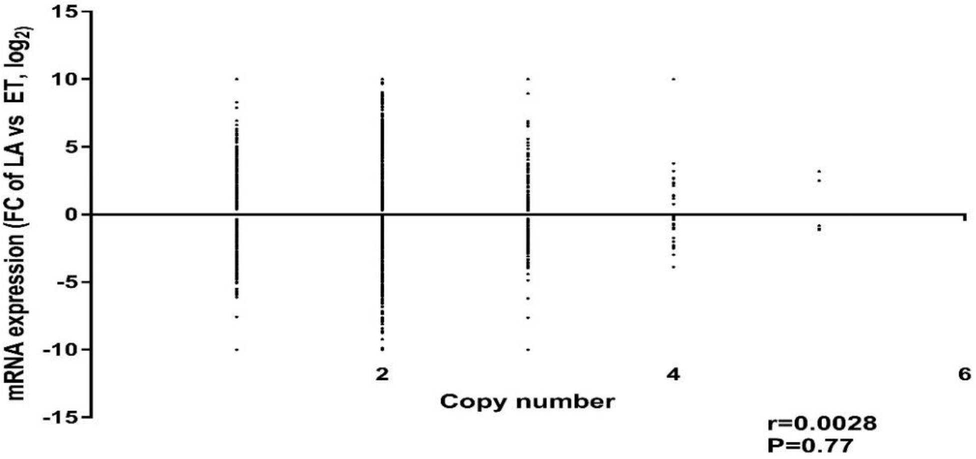

Supplementary Figure S4. Inter-data relationships of CNV with differential RNA-seq analysis in PMC42 cell lines (A) Log_2 FC (fold change) of differential mRNA expression levels were linked to the genomic copy number and spearman's correlation coefficient (*r* with *p*-value) is indicated at the bottom right.
